# P-342. Persistent Low-Level Viremia is Associated with Virologic Failure Even Among People With HIV (PWH) Initiating Single Tablets Antiretroviral Therapy (ART) Regimens

**DOI:** 10.1093/ofid/ofaf695.560

**Published:** 2026-01-11

**Authors:** Anuradha Ganesan, Hsing-Chuan Hsieh, Christie Joya, Tahaniyat Lalani, Rhonda E Colombo, Joseph Yabes, Derek T Larson, Jason Blaylock, Brian Agan

**Affiliations:** Uniformed Services University of Health Sciences, Bethesda, Maryland; Infectious Disease Clinical Research Program, Bethesda, Maryland; Naval Medical Center Portsmouth, Portsmouth, Virginia; Naval Medical Center Portsmouth, Portsmouth, Virginia; Infectious Disease Clinical Research Program, USUHS; Henry M. Jackson Foundation for the Advancement of Military Medicine, Inc., Bethesda, Maryland; Brooke Army Medical Center, San Antonio, Texas; Naval Medical Center San Diego and Uniformed Services University, SAN DIEGO, CA; Walter Reed National Military Medical Center, Bethesda, Maryland; Infectious Disease Clinical Research Program, Department of Preventive Medicine and Biostatistics, Uniformed Services University of the Health Sciences, Bethesda, MD, USA, Bethesda, Maryland

## Abstract

**Background:**

Persistent Low-Level Viremia (pLLV) has been associated with Virologic Failure (VF). Use of Single Tablet ART Regimens (STRs) is associated with improved adherence and virologic outcomes. The prevalence and implications of LLV amongst PWH initiating STRs is not well-studied. We examined virologic implications of LLV in those initiating STRs in the US military HIV Natural History Study (NHS).

**Figure 1.**
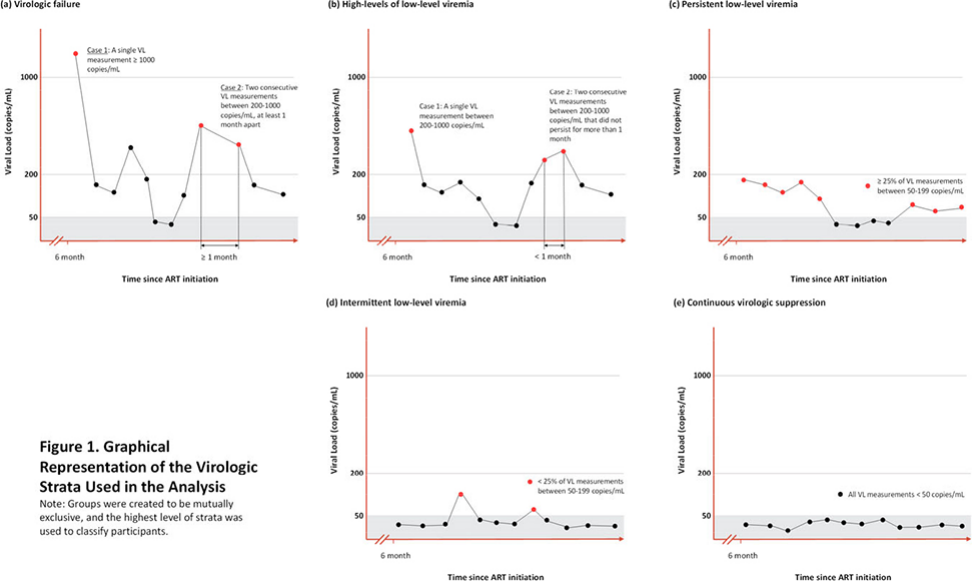
Graphical Representation of Virologic Strata used in the analysis

**Table 1:**
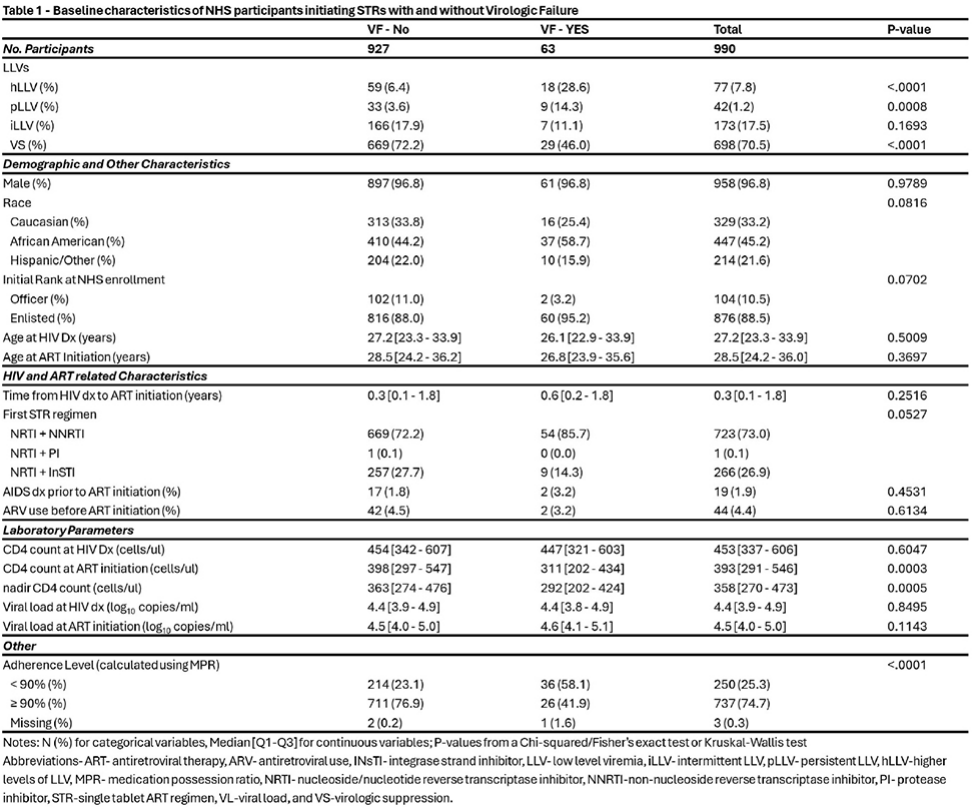
Baseline Characteristics of NHS participants initiating STRs With and Without Virologic Failure

**Methods:**

NHS participants have unrestricted access to care and medications, minimizing the barriers to achieving and maintaining virologic suppression (VS); included participants had ≥2 viral loads (VL) measured with an assay with a lower limit of detection of ≤50 copies/ml, 6 months post STR initiation. VF was defined as a VL ≥200 copies/mL on 2 consecutive determinations spanning at-least 30 days or any VL ≥1000 copies/mL after VS. Participants were categorized into mutually exclusive LLV categories: intermittent LLV (iLLV) (VL of 50–199 copies/mL on < 25% of measurements), pLLV (VL of 50–199 copies/mL on ≥25% of measurements), high-levels of low level viremia (hLLV) VL of 200–1000 copies/mL) that did not meet criteria for VF, and VS (all VL < 50 copies/mL), Figure 1. VL measurements during ART interruptions were not included. Participants were censored at last follow- up or at VF. Adjusted time updated Cox proportional hazards models were used to evaluate the association between prespecified risk factors and VF.

**Table 2:**
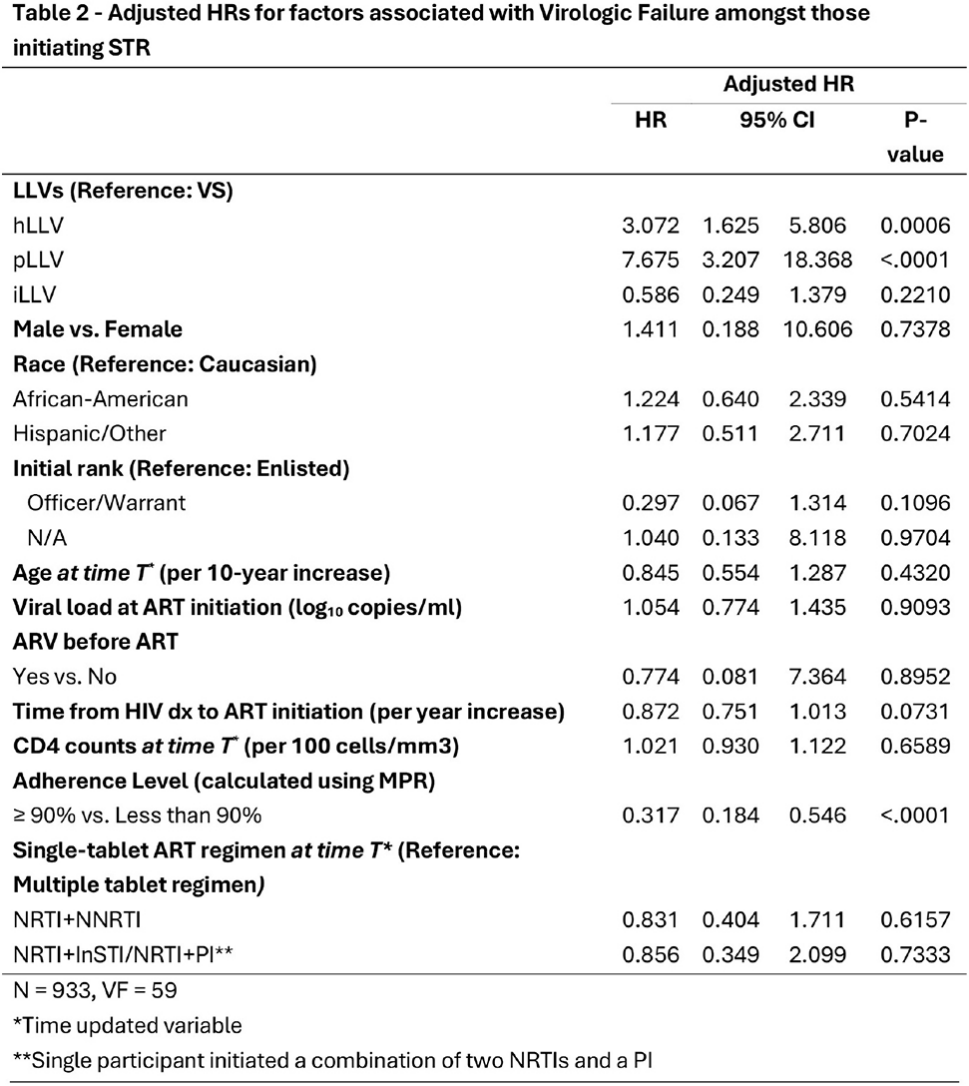
Adjusted Hazard Ratios for Factors Associated with Virologic Failure Amongst Those Initiation Single Tablet ART Regiments

**Results:**

A total of 990 participants (median age,28.5 years at STR initiation, 97% male and 45% African American) contributed a median of 5.8 years follow up and 9 VL measures; 63 participants (6.4%) experienced VF, Table 1. A total of 292 (26.6%) participants experienced LLV: pLLV (n=42, 1.2%), iLLV (n=173, 17.6%), or hLLV (n=77, 7.8%). Both pLLV (adjusted HR (aHR) 7.7 [95% CI: 3.2 – 18.4)] and hLLV were associated with VF (aHR 3.1 [95% CI: 1.6 – 5.8]). VF was also associated with suboptimal adherence, characterized by medication possession ratios of < 90%, Table 2.

**Conclusion:**

LLV was common in participants initiating STRs, about 1 in 4 participants had one or more episodes. Even after adjusting for adherence, both pLLV and hLLV were associated with VF. Our observations suggest that pLLV is more than just a random phenomenon and argue for closer clinical monitoring of PWH with pLLV and hLLV on STRs.

**Disclosures:**

All Authors: No reported disclosures

